# Supplementary material for: Yiai Fuzheng decoction inhibits triple-negative breast cancer by remodeling the immune microenvironment
Source: Front Immunol. 2025 Sep 30;16:1615631. doi: 10.3389/fimmu.2025.1615631 (PMC12518410; doi:10.3389/fimmu.2025.1615631)
Supplement: Supplementary file 2 [file DataSheet1.zip › Supplementary File 2/Treatment/C_vs_D/07_Pathway_Analysis/Enrichment_Analysis/predicted/Enrichment_Barplot.pdf]

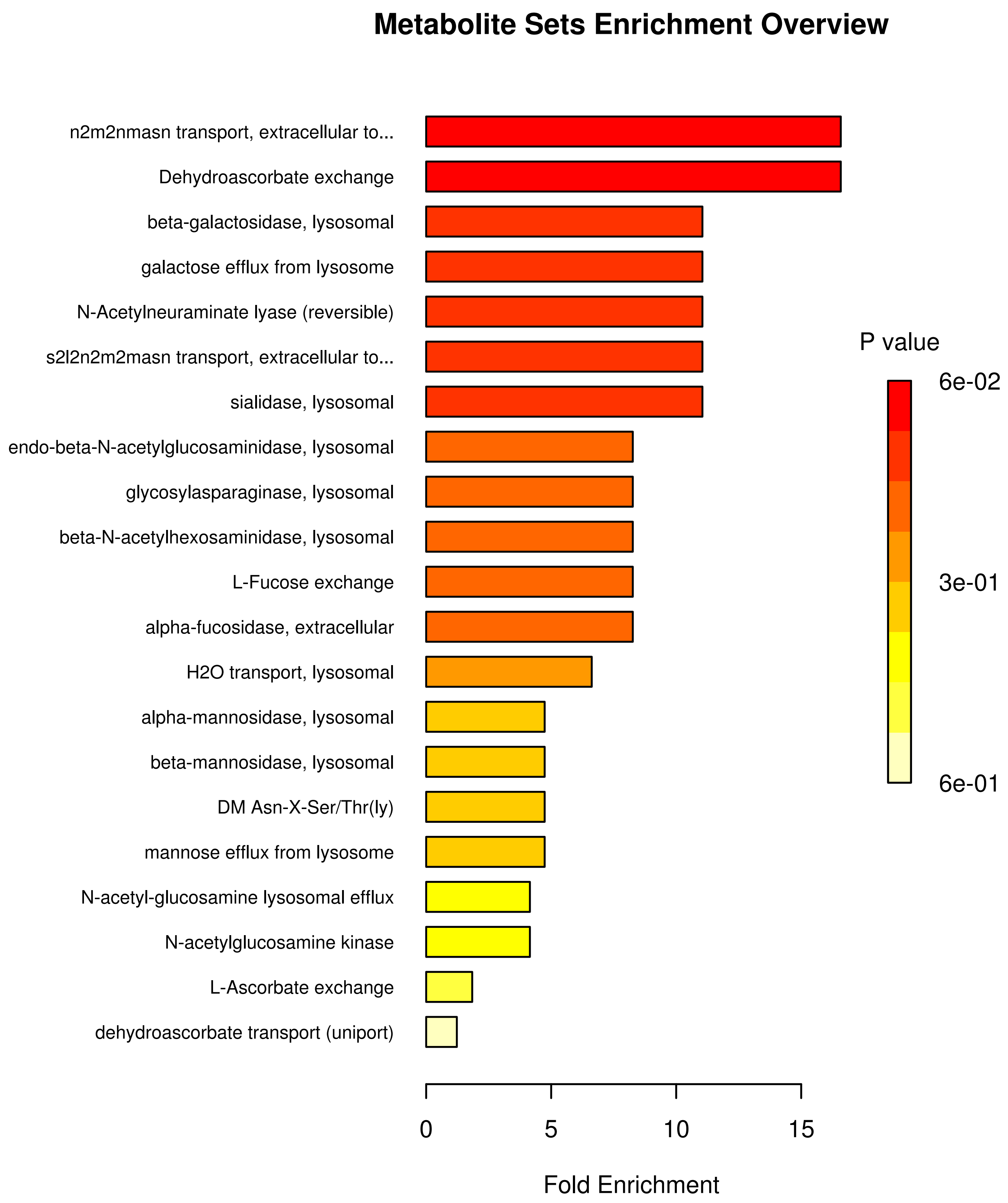
Metabolite Sets Enrichment Overview
n2m2nmasn transport, extracellular to...
Dehydroascorbate exchange
beta-galactosidase, lysosomal
galactose efflux from lysosome
N-Acetylneuraminate lyase (reversible)
s2l2n2m2masn transport, extracellular to...
sialidase, lysosomal
endo-beta-N-acetylglucosaminidase, lysosomal
glycosylasparaginase, lysosomal
beta-N-acetylhexosaminidase, lysosomal
L-Fucose exchange
alpha-fucosidase, extracellular
H2O transport, lysosomal
alpha-mannosidase, lysosomal
beta-mannosidase, lysosomal
DM Asn-X-Ser/Thr(ly)
mannose efflux from lysosome
N-acetyl-glucosamine lysosomal efflux
N-acetylglucosamine kinase
L-Ascorbate exchange
dehydroascorbate transport (uniport)
P value
6e-02
3e-01
6e-01
0
5
10
15
Fold Enrichment
